# IDH Mutation Assessment in Gliomas from Anatomical MRI Using Deep Learning: A Comparative Analysis of Centralized and Federated Learning Frameworks

**DOI:** 10.3390/diagnostics16040623

**Published:** 2026-02-20

**Authors:** Abdullah Bas, Esin Ozturk-Isik

**Affiliations:** 1 Institute of Biomedical Engineering, Bogazici University, Istanbul 34684, Türkiye; esin.ozturk@bogazici.edu.tr; 2 Center for Targeted Therapy Technologies, Bogazici University, Istanbul 34684, Türkiye

**Keywords:** isocitrate dehydrogenase (IDH), glioma, MRI, post-contrast T1-weighted, T2-weighted, FLAIR, federated learning

## Abstract

**Background/Objectives:** Isocitrate dehydrogenase (IDH) mutation is a key prognostic indicator in diffuse gliomas; however, it is clinically determined from invasive tissue sampling. Non-invasive preoperative identification of IDH mutation from routine anatomical MRI could support treatment decision making. This study evaluated deep learning models for IDH mutation detection using routine anatomical MRI (post-contrast T1-weighted (T1c), T2-weighted, and fluid attenuated inversion recovery (FLAIR) MRI) and quantified how tumor-focused image preprocessing and different training schemes, centralized learning (CL) versus federated learning (FL) with alternative aggregation strategies, affected model performance. **Methods:** Anatomical MRI from 501 diffuse glioma patients in the UCSF Preoperative Diffuse Glioma MRI (UCSF-PDGM) dataset was analyzed using a deep learning classifier built on a 2D U-Net encoder, with age and sex included as covariates. Two methods of tumor-focused image preprocessing, Naïve Soft Filtering (NSF) and Gradient-Based Soft Filtering (GBSF), were compared. Centralized learning (CL) was benchmarked against federated learning (FL) using Federated Averaging (FA) and Federated Trimmed Mean (FTM) aggregation strategies. Model performance was compared in terms of accuracy, precision, recall, F1 score, specificity, and the area under the receiver operating characteristic curve (ROC-AUC). **Results:** The CL model with NSF achieved the best test performance (accuracy = 0.949, F1 = 0.951, ROC-AUC = 0.971), with NSF consistently outperforming GBSF. FL’s performance decreased relative to CL’s, but the FA strategy outperformed FTM (FTM accuracy = 0.915 vs. FA accuracy = 0.949), which indicates that the FL aggregation strategy has an influence on model performance. **Conclusions:** Deep learning applied to routine anatomical MRI could classify IDH mutation status with high accuracy. Context-preserving image preprocessing with NSF substantially improved performance across training schemes. FL provides a privacy-preserving alternative to CL, but incurs a measurable performance degradation that is sensitive to the choice of aggregation strategy.

## 1. Introduction

Isocitrate dehydrogenase (IDH) mutation status is a key molecular determinant in gliomas, with implications for prognosis, therapeutic planning, and clinical trial stratification. The fifth edition of the World Health Organization’s (WHO) classification of central nervous system tumors (WHO CNS5) was the second guideline, after WHO 2016, that reinforced the significance of IDH mutations for classifying gliomas [1,2,3,4]. Besides IDH mutations, 1p/19q codeletion, H3F3A mutations, ATRX mutations, and MGMT promoter methylation were also highlighted for glioma classification in WHO CNS5.

IDH mutant (IDH-mut) gliomas generally exhibit a more favorable prognosis than IDH-wildtype (IDH-wt) gliomas. Although some studies have reported no significant prognostic differences between IDH-wt and IDH-mut gliomas [5], many studies have reported better prognosis for IDH-mut gliomas [6,7,8,9,10,11,12,13,14]. Moreover, one study reported a statistically significant relationship between IDH mutation status and mitotic index [15]. There has been recent progress in treating IDH-mut gliomas, underlying its importance in patient management. A recent clinical trial, INDIGO, has shown that vorasidenib, a small-molecule inhibitor targeting the IDH1 and IDH2 enzymes, significantly improved progression-free survival in IDH-mut gliomas [16], and it was approved by the FDA in 2024. Anatomical MRI-based noninvasive estimation of IDH mutational status thus meets a significant clinical need in this regard, especially with the advent of targeted treatments for IDH-mut gliomas.

Due to the limitations of tissue sampling owing to the heterogeneity of tumors and surgical risk, non-invasive preoperative prediction of IDH status in terms of deep learning (DL) models based on MRI data is of great interest. Initial convolutional models proved that conventional anatomic differences have informative representations. A T2-weighted (T2w) and MC-Net dual-network CNN model (post-contrast T1-weighted (T1c) and fluid-attenuated inversion recovery (FLAIR)) yielded cross-validated accuracies >90% and revealed that T2w MRI in particular provided important information for detecting IDH-associated variability [17]. In a cohort of 495 patients, Elyassirad et al. [18] compared 2D and 3D ResNet architectures and found a transfer-learning-based 2D ResNet-50 to be superior to the 3D models (AUC = 0.91), underscoring the practicality of 2D backbones for clinical MRI.

Transformer models and multi-modal feature integration have further advanced performance and interpretability. A Vision Transformer (ViT) with masked-autoencoder self-pretraining operating on whole-slice T1c, T2, and FLAIR attained 93% internal accuracy, and Grad-CAM maps localized predictions to T2-hyperintense and necrotic regions, offering biologically plausible attributions [19]. Incorporating microstructural diffusion features can add complementary signals, and using VGG16 as a feature extractor on anatomic MRI plus diffusion tensor imaging (DTI) maps increased sensitivity to 91.1% [20]. Beyond single-task prediction, a multi-task deep model jointly learned IDH mutational status, prognosis, and molecular subtypes, linking imaging phenotypes with pathways such as epithelial–mesenchymal transition via radio-multiomics (AUC = 0.89–0.90) [21]. Related groundwork in automated tumor segmentation with nnU-Net across 1685 patients (T1c, T2-FLAIR, apparent diffusion coefficient (ADC)) reported AUC = 0.948 for segmentation and identified age as an influential covariate for downstream modeling [22].

Synthesis of this literature via meta-analysis (52 studies) indicated pooled sensitivity/specificity of 0.84/0.87 (AUC = 0.89) for MRI-based IDH classification, while also emphasizing substantial heterogeneity in MRI protocols, segmentation strategies, and validation designs [23]. Methodologically, deep learning-based radiomics eliminates manual feature crafting by extracting large-scale deep features directly from images (e.g., 16,384 features in a modified CNN), streamlining pipelines and potentially improving generalizability [24].

Overall, the literature supports robust and interpretable MRI-based classification of IDH mutational status using CNNs and transformers, especially when multi-contrast inputs and automated segmentation are leveraged. Nonetheless, protocol heterogeneity, limited external validation, and variable image preprocessing continue to impede broad clinical adoption. Dataset variability remains a central obstacle, and deep models often lose accuracy when deployed across sites due to image domain shift. This shift arises from differences in MRI protocols (for example TR/TE, field strength, and coil configuration), vendor-specific reconstruction, preprocessing pipelines, and population demographics. As a result, external validation typically underperforms compared to internal validation. A meta-analysis of 11 studies (n = 1685) reported substantial performance heterogeneity, with AUCs ranging from 0.74 to 0.98 [25]. Even models with strong internal results can show external drops, as in Yu et al. [19], where a ViT achieved 93% internal accuracy but 87% on an external cohort. A key contributor to this variability is the scarcity of multi-institutional training, constrained by data-sharing and privacy regulations (for example, General Data Protection Regulation (GDPR) and the Health Insurance Portability and Accountability Act (HIPAA)) that limit centralized pooling, reduce cohort diversity, and weaken cross-site generalization. Federated learning (FL) offers a practical alternative by training models locally on site-resident data and sharing only weight updates or gradients for server-side aggregation, thereby enabling learning from distributed, diverse cohorts while reducing scanner and region-specific bias.

In this study, we quantified the performance gap between centralized and federated learning for IDH mutation status identification in gliomas under controlled conditions, using pseudo-site partitions within a single data cohort. By holding acquisition and demographic factors effectively constant, the analysis isolated the intrinsic costs of federation from confounds related to cross-site domain shift. Centralized and federated models were benchmarked with harmonized code and matched image preprocessing, two federated aggregation rules were compared, and two tumor-focused filtering strategies were evaluated. This design provides actionable guidance on when federated learning can approach centralized performance and which design choices help preserve external validity in IDH classification pipelines, with direct relevance to routine clinical MRI.

## 2. Materials and Methods

### 2.1. Materials

This study analyzed preoperative MRI data from 501 diffuse glioma cases (age range 17–94, mean 56.87 ± 15.02; 298 males, 203 females, 103 IDH-mut, 398 IDH-wt), originally sourced from the University of California San Francisco Preoperative Diffuse Glioma MRI (UCSF-PDGM) dataset [26] (Table 1). IDH mutation status was confirmed by either conventional or next-generation sequencing of tumor tissue. MRI data were acquired on a 3T clinical MRI scanner (GE Healthcare, Waukesha, WI, USA) using a standard brain tumor imaging protocol including post-contrast T1-weighted inversion-recovery spoiled gradient echo (IR-SPGR, TR = 6 ms, TE = 2.3 ms, TI = 450 ms, flip angle = 12°), 3D T2-weighted fast spin echo (TR = 2200 ms, TE = 100 ms), and FLAIR (TR = 5700 ms, TE = 115 ms, TI = 1650 ms).

The MRI data of each patient were resampled to a 1 mm^3^ isotropic resolution and co-registered to 3D space defined by the T2/FLAIR using nonlinear registration via ANTs. Skull stripping was performed using a publicly available deep learning-based brain mask algorithm [27]. Skull-stripping and tumor segmentations were obtained from the data publishers and visually inspected by the authors prior to use. Multiple-compartment tumor segmentations (enhancing tumor, non-enhancing/necrotic core, peritumoral FLAIR abnormality) performed using an ensemble of BraTS-derived models, with manual correction by at least two expert reviewers with more than 15 years of experience, were also provided with the dataset [26].

**Figure 1:**
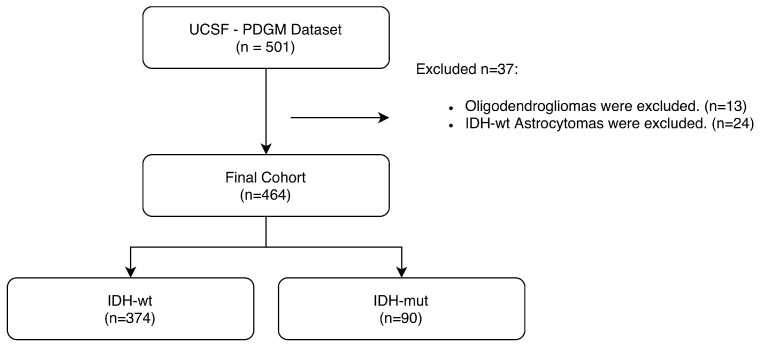
Patient cohort distribution in the UCSF-PDGM dataset and applied exclusion criteria.

The UCSF-PDGM dataset is dominated by glioblastomas (IDH-wt; n = 398) and contains a highly imbalanced number of oligodendrogliomas (IDH-mut, 1p/19q co-deleted, n = 13). Given this extreme class imbalance, oligodendrogliomas were excluded to avoid introducing bias and instability into the reported results. In addition, the dataset includes 24 astrocytomas, labeled as IDH-wt, without any accompanying molecular markers. Due to incomplete molecular characterization and the resulting diagnostic ambiguity, IDH-wildtype astrocytomas were excluded to comply with the WHO 2021 classification requirements. The patient cohort distribution and the exclusion criteria are shown in (Figure 1).

### 2.2. Methods

**Figure 2:**
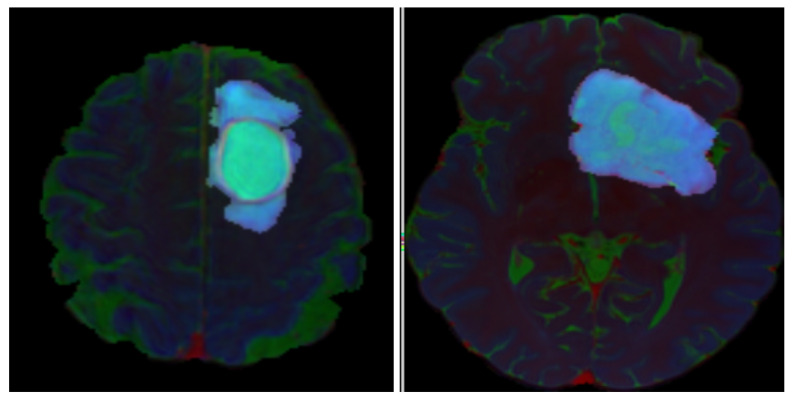
Two example RGB image slices that belong to an IDH-wildtype. (**left**) and an IDH-mutant (**right**) glioma from the test dataset of Naïve Soft Filtering. R: FLAIR, G: T1c, B: T2w.

**Figure 3:**
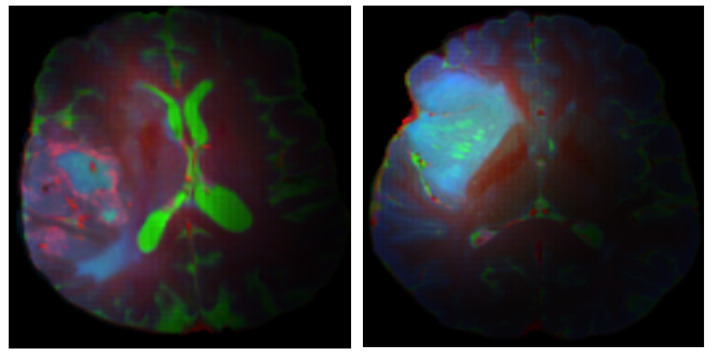
Two example RGB image slices that belong to an IDH-wildtype. (**left**) and an IDH-mutant (**right**) glioma from the test dataset of Gradient-Based Soft Filtering. R: FLAIR, G: T1c, B: T2w.

For each patient, a comprehensive tumor region-of-interest (ROI) mask was generated by integrating segmentations of enhancing tumor, FLAIR abnormality, and necrotic regions. To prioritize tumor-related features while retaining contextual anatomical information, two soft filtering approaches were evaluated to preserve the spatial context of the MR images while emphasizing tumor regions. In the first approach, we applied a fixed-weight soft filtering scheme (Naïve Soft Filtering; NSF) in which non-tumorous regions outside the tumor borders were assigned a heuristically selected weight of 0.3 during both training and testing. This weighting ensured that these regions contributed partially to the loss function (Figure 2). The factor of 0.3 was kept constant across all experiments to maintain consistency and comparability. In the second approach, we introduced a Gradient-Based Soft Filtering strategy, which assigned spatially varying weights that gradually decreased from 0.3 near the tumor boundary to 0 at the farthest points of the image (Gradient-Based Soft Filtering, GBSF) (Figure 3). This approach collected more contextual information near the tumor while suppressing the influence of regions with respect to the distance from the tumor. For axial slices containing tumor tissue, three MRI sequences, FLAIR, T1c, and T2w, were mapped to the red, green, and blue channels of an RGB image, respectively. This color-space transformation leveraged the complementary contrast properties of each sequence, in which FLAIR (red) highlighted edema and non-enhancing tumor, T1c (green) delineated enhancing tumor regions, and T2w (blue) captured overall tumor morphology. Each channel underwent individual intensity normalization (zero-mean, unit variance) to mitigate scanner-specific variations.

**Figure 4:**
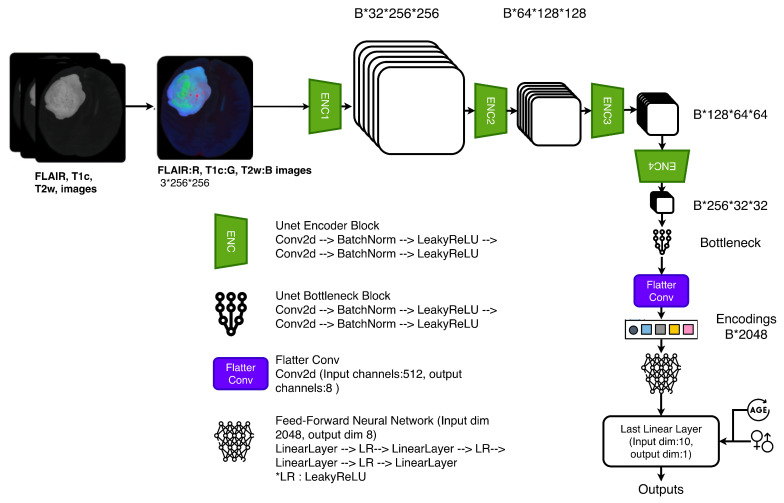
The classifier network architecture.

The classifier network architecture employed a 2D U-Net encoder pretrained on tumor segmentation tasks to extract spatially resolved features, followed by task-specific convolutional layers for IDH classification (Figure 4). The U-Net encoder was pretrained on an independent in-house glioma dataset, with no patient overlap and no molecular labels used during pretraining. Additionally, both datasets underwent standard preprocessing (skull-stripping, registration, intensity normalization), and encoder weights were fine-tuned rather than frozen during classification training to allow adaptation to potential domain differences while leveraging pretrained anatomical feature representations. This design leveraged U-Net’s ability to capture multi-scale contextual features while minimizing computational overhead compared to 3D approaches.

The network consisted of multiple encoder layers following the standard U-Net [28] encoder design. Each encoder layer (ENC) consisted of two 2D convolutional layers, two batch normalization layers, and two leaky ReLU activation layers. The bottleneck block, which reduced the channel dimension from 1024 to 512, used the same layer types as the encoder but with different configurations. The Flatter, a simple 2D convolutional layer, reduced the channels from 512 to 8, decreasing the number of features after flattening. A feed-forward neural network was added to process the flattened features to obtain a scalar output for identifying the IDH mutational status. On the last linear layer, age and sex were also added to boost the performance of the model. Each model was trained with an AdamW optimizer (learning rate = 1 × $10^{-4}$) and a cosine annealing learning rate schedule (T_max = 10, n_min = 1 × $10^{-6}$). Centralized models were trained on aggregated data for 40 epochs. In federated learning experiments, a single local epoch was performed on each model per round, followed by server aggregation. The total number of rounds was chosen to ensure an equal optimization budget for federated and centralized experiments. Early stopping was not used.

**Figure 5:**
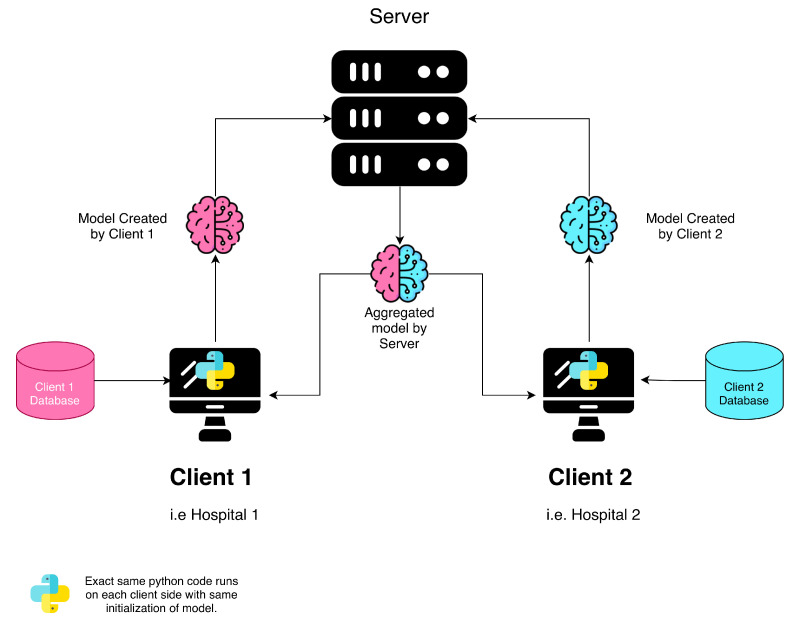
A schematic representation of the FL architecture.

The federated learning (FL) framework was implemented using Flower [29], a scalable library for decentralized model training. The dataset, consisting of 464 patients, was partitioned into a training cohort (n = 361), a validation cohort (n = 44), and a holdout test set (n = 59). For centralized learning (CL), the entire training set was used. In the FL paradigm, the training data were evenly distributed between two clients, with careful balancing to ensure comparable IDH mutation ratios (IDH-mutant: around 15% in both splits; (Table 2)). To evaluate FL robustness, two aggregation strategies were tested: (1) Federated Averaging (FA), which computes a simple mean of client model weights, and (2) Federated Trimmed Mean (FTM), which discards outlier parameters to enhance resilience against non-independent and identically distributed (non-IID) data distributions. Identical test sets and model seeds (seed = 61) were used across all experiments to isolate the impact of FL versus centralized training. Figure 5 shows the FL scheme. Weights & Biases (W&B) was used for real-time monitoring of training dynamics and performance [30]. At each optimization step, training/validation loss and the full evaluation suite of accuracy, precision, recall, F1 score, specificity, and ROC-AUC were logged, enabling fine-grained inspection of learning behavior and supporting reproducibility through centralized experiment tracking.

## 3. Results

Table 3 reports the performance of the proposed CL and FL approaches for IDH-mutation classification in gliomas. Test-set class proportions were intentionally balanced to avoid majority-class bias in threshold-dependent metrics. As a result, precision-recall values reflect performance under balanced prevalence and may differ under natural class distributions, whereas prevalence-insensitive metrics such as ROC-AUC, and specificity provide more generalizable assessments of discriminative ability. The CL model with NSF achieved the highest overall performance, with accuracy = 0.949, F1 = 0.951, and ROC-AUC = 0.971. This configuration provided a favorable precision-recall balance (precision = 0.935; recall = 0.966) and high specificity (0.931), indicating effective detection of IDH-mutant cases.

**Figure 6:**
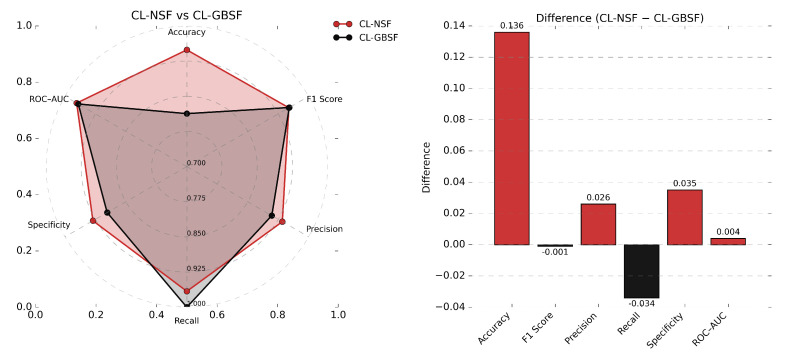
Comparison of CL’s performance on different datasets. The radar plot on the left hand side shows a comparison of CL models that were trained on the two different data generation strategies. The bar plot on the right hand side shows the performance differences between those two models.

Under CL with GBSF, the model achieved an accuracy of 0.813 for IDH-mutation classification. Precision (0.952) was significantly higher than recall (0.667), in contrast to the CL model trained with NSF. The resulting F1 score of 0.784 reflected an imbalanced precision-recall trade-off, suggesting class-specific bias under the observed class distribution. The specificity was 0.966, indicating relatively few false positives. Figure 6 illustrates the performance differences in terms of metrics between the two configurations.

Given the superior performance of the CL models on data generated by the NSF approach, the FL models were trained only on NSF-generated data. Using the FTM aggregator, the model achieved accuracy = 0.915, precision = 0.963, recall = 0.867, F1 = 0.912, specificity = 0.966, and ROC-AUC = 0.957. The higher precision than recall, together with the specificity of 0.966, indicates fewer false positives than false negatives.

Using FA, the FL model achieved accuracy = 0.949, F1 = 0.952, precision = 0.909, recall = 1.000, specificity = 0.896, and ROC-AUC = 0.967. In contrast to FTM, recall exceeded precision, indicating fewer false negatives than false positives. This precision–recall pattern matched the CL model trained on NSF-generated data.

**Figure 7:**
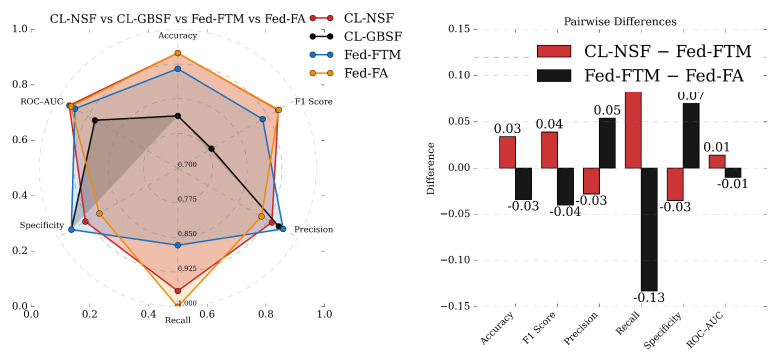
Comparison of CL’s and FL’s performance on different datasets. The radar plot on the left hand side shows a comparison of the CL and FL models that were trained on different data generation strategies. The bar plot on the right hand side shows the performance differences between CL and FL models trained on NSF dataset, and FL models with different aggregation strategies.

Figure 7 summarizes the performance metrics for the CL and FL strategies and their pairwise differences. The left-hand side reports results for the CL model trained on NSF data, alongside both FL variants (FTM and FA). The right-hand side plot shows the metric deltas relative to the CL baseline, highlighting how the choice of FL strategy affects outcomes. Notably, the magnitude of the differences between FTM and FA underscores that the federated aggregation rule is a first-order design choice rather than a secondary detail.

The radar plot on the left hand side shows the performance of each model, while the figure on the right hand side shows the performance differences between different models.

**Figure 8:**
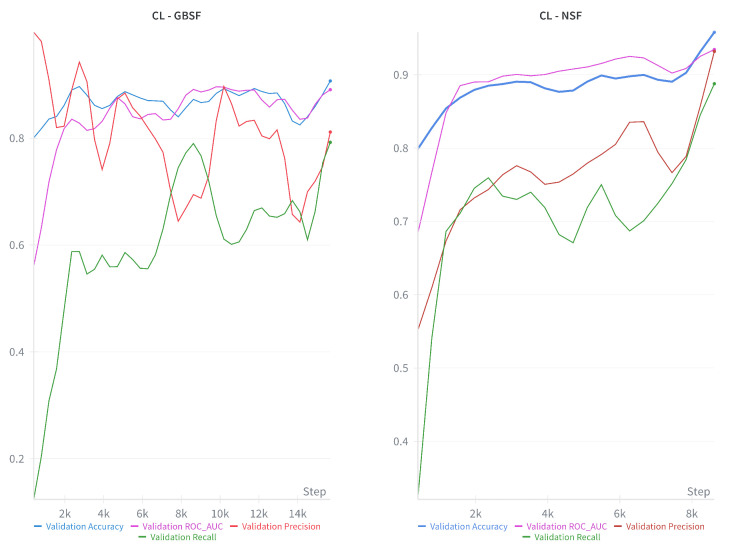

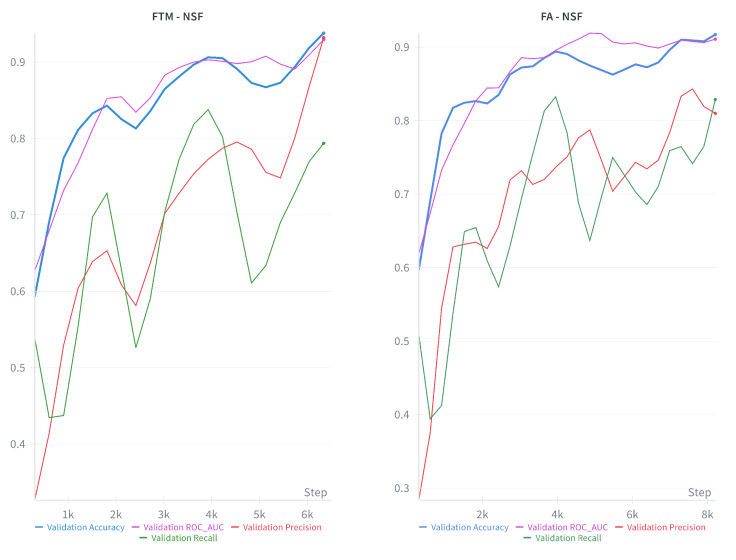
Performance trajectories of all models during validation. The monitoring graphs show the performance metrics of CL trained on the data generated with GBSF (**top left**) and NSF (**top right**), and FL-FTM (**bottom left**) and FL-FA (**bottom right**) trained on the data generated by NSF.

In Figure 8, the monitoring results obtained using the W&B monitoring tool are shown [30]. All the metrics that are displayed were observed on the validation set of the study. Figure 8 is intended to provide qualitative insight into the training dynamics rather than to serve as a direct quantitative comparison of metric performances. Consistently across experiments, CL exhibits more stable convergence and superior overall performance compared to FL, which shows higher volatility. Within federated methods, the FA strategy demonstrates greater stability than FTM. It is important to note that these plots have been smoothed to enhance the visibility of general convergence trends; consequently, they should be interpreted as identifying behavioral patterns rather than recording exact performance values at specific steps.

## 4. Discussion

This study evaluated MRI-based detection of IDH mutation status in gliomas using only preoperative, routine anatomical MRI sequences (T1c, T2, FLAIR). Multiple training paradigms using centralized learning versus federated learning and tumor-focused preprocessing strategies (NSF versus GBSF) were compared. The CL model trained on pooled data with NSF yielded the strongest overall performance, suggesting that context-preserving attenuation around the lesion may be beneficial for discrimination under the evaluated conditions, without sacrificing specificity or recall. The FL model with the FA aggregator achieved slightly less imbalanced performance in contrast to CL but offered a data-sensitive approach that better accounts for site-specific variability.

Under centralized learning, the model that trained on GBSF data underperformed compared to its NSF counterpart. This degradation may be related to differences in how tumor subregions are emphasized by the spatial weighting scheme; however, direct attribution analyses would be required to confirm the contribution of the intratumoral signal to IDH mutation detection. In other words, prioritizing boundary voxels may have diluted informative core features and destabilized feature learning. Under FTM, performance decreased relative to CL, consistent with training on disjoint, site-partitioned data. Because each client model was exposed only to its local subset and aggregated updates (rather than pooled data), the global model likely suffered from non-IID effects and limited cross-site feature alignment. These findings underscore the benefit of training on pooled data, which affords broader feature diversity and, in this cohort, translated into superior discrimination compared with FL variants.

A key methodological observation was that the choice of FL aggregation strategy influenced performance, with differences between FL variants approaching the magnitude of the CL-to-FL transition itself. FA outperformed FTM in terms of accuracy. The largest divergence occurred in recall, indicating markedly more false negatives under FTM, which is an especially concerning error mode for clinical decision-making. Furthermore, although FTM is designed to be robust to outlier updates, in this setting it appears to suppress informative site-specific variability, leading to a marked increase in false negatives. This trade-off indicates that robustness-oriented aggregation can inadvertently reduce recall when inter-site differences reflect meaningful clinical heterogeneity rather than noise.

Ablation results highlighted the value of peri-tumoral context. Retaining non-lesional regions with NSF, which attenuates rather than excises background, preserved cues related to edema, mass effect, and tissue interfaces and corresponded to the strongest overall performance. In contrast, a gradient-based scheme that emphasized the lesion core increased precision at the expense of recall, yielding more false negatives. This precision–recall trade-off is clinically critical. Core-emphasizing filters may be preferable in settings where missing a positive case carries higher risk, such as pre-surgical triage. Context-preserving attenuation is advantageous when minimizing unnecessary follow-up is the primary goal.

In the literature, some studies have investigated tumor detection and biomarker analysis using T1 or multimodal MRI, demonstrating the potential of radiological imaging combined with machine learning. However, these works differ substantially from the present study in terms of input data, methodological design, and analytical focus. Although prior approaches typically rely on advanced radiomic feature extraction or explicit segmentation pipelines, our work uses routine anatomical MRI with tumor-focused preprocessing. Critically, rather than optimizing performance on a single diagnostic task, we evaluate model generalizability and aggregation effects across different training paradigms, which represents a distinct and complementary contribution to the field [31,32].

Furthermore, several prior studies have reported strong results for MRI-based IDH classification. Usuzaki et al. [33] combined radiomics features with MR images to train a ViT model and achieved 93.5% accuracy on external verification, noting that radiomics features contributed most strongly to discrimination. Other reports on different datasets illustrated that headline accuracy can mask class imbalance. For example, one study reported 96% accuracy with an F1 score of 75%, suggesting skewed class distributions and limited positive-class recall [34]. Another investigation using perfusion MRI with recurrent neural networks achieved 92.8% accuracy for IDH classification [35]. A hybrid approach that fused radiomics with T1 and T2w MRI attained 93.8% internal accuracy and 87.9% and 78.8% on two external cohorts, highlighting the challenge of cross-site generalization [36]. Bjørkeli and Esmaeili [37] combined T1, T1c, FLAIR, and T2 MRI, and reported 97.6% test accuracy for IDH and 1p/19q codeletion classification, but sensitivity was 56.9%, again indicating imbalance and a high false-negative rate. Finally, a two-center comparison of centralized learning and federated variants reported 83.13% accuracy under centralized training, 81.96% with vanilla federated learning, and up to 83.37% with an alternative federated strategy [38], underscoring the impact of optimization choices and data distribution on performance.

On the other hand, compared with prior studies that relied on radiomics features, diffusion or perfusion imaging, or specialized multi-omics pipelines, this study evaluated IDH classification using only routine preoperative anatomical MRI (T1c, T2w, FLAIR) within a single, unified deep learning framework. Additionally, age and sex were incorporated as covariates at the final classification layer by concatenating them with the imaging-derived features, motivated by prior evidence that these factors are associated with IDH mutation status [11,39]. Moreover, the study design isolated three practical factors that are rarely examined together: training paradigm, aggregation strategy in federated settings, and tumor-focused filtering. First, centralized learning was directly compared with federated learning using harmonized code, identical seeds, and matched image preprocessing to quantify the generalization cost of distributing data across sites. Second, two federated aggregation rules were contrasted on the same cohorts, showing that the choice of aggregation strategy produced performance shifts, a result that emphasizes optimization policy as a primary design variable rather than a minor implementation detail. Third, NSF was evaluated against a gradient-based scheme to characterize the precision–recall trade-offs introduced by preserving peri-tumoral context versus emphasizing the lesion core. Taken together, the contribution of this study is a clinically oriented and systematically controlled analysis that clarifies how deployment-relevant choices regarding training paradigm, aggregation, and filtering shape IDH classification performance when only standard clinical MRI is available.

This study has some limitations. Only routine anatomical MRI was used, and advanced MRI modalities such as perfusion, diffusion, and MR spectroscopy were not incorporated. Although this has a realistic clinical baseline and can be considered an advantage for deployability, it reduced the model’s ability to capture microstructural, hemodynamic, and metabolic signatures and limited the data content to a narrower anatomical view. Another limitation is the use of a single publicly available glioma MRI dataset (UCSF-PDGM). Although 1p/19q codeletion status is a critical molecular marker for integrated glioma classification, it was not included in this study due to data limitations. The UCSF-PDGM dataset contains only 13 1p/19q codeleted oligodendrogliomas out of 501 total cases, representing severe class imbalance (only 2.6% 1p/19q codeleted) that would preclude reliable statistical analysis or model training for including this molecular marker. Access to larger datasets with complete molecular annotation would enable broader application of federated learning for integrated glioma classification, including 1p/19q status and other biomarkers. Furthermore, limiting training and evaluation to one source may have reduced cross-site heterogeneity and, in turn, may have inadvertently enhanced the apparent performance of the federated methods by minimizing real-world domain variability. However, by constructing pseudo client splitting within this cohort and keeping acquisition and demographic factors constant, the analysis isolated the internal performance costs of FL, independent of cross-institutional image domain shift. This design clarified FL’s baseline trade-offs, although the single-center scope remains a limitation for external generalizability. Additionally, FL has important constraints, and data distributions, label imbalance, and unequal client sizes can induce client drift and unstable convergence. As a result, additional safeguards and harmonization steps are required for multi-institutional data, including secure aggregation and differential privacy to protect shared updates, as well as site-aware normalization such as intensity standardization, bias-field correction, or statistical harmonization. Personalized FL strategies, such as site-specific batch normalization or lightweight local adaptation and ensemble-style aggregation, can further stabilize training and improve robustness. Since our experiments used partitions created from a single-site dataset, future studies on this topic will need to integrate these optimizations to ensure robustness under real-world multi-center conditions.

## 5. Conclusions

In conclusion, deep learning models using routine anatomical MRI can classify IDH mutations in gliomas with high accuracy. CL with NSF achieved the best overall performance, while federated learning remained a viable privacy-preserving alternative, whose effectiveness depended strongly on the aggregation strategy. Data preprocessing choices shaped the error profile. GBSF favored precision, whereas NSF preserved peri-tumoral context and improved overall discrimination. Future work should validate these findings in multi-institutional cohorts and explore multimodal inputs and adaptive filtering strategies within federated training frameworks.

## Figures and Tables

**Table 1:** The patient characteristics of the UCSF-PDGM dataset.

Features	Total (n = 501)	IDH-mut (n = 103)	IDH-wt (n = 398)
Age (years)	56.87 ± 15.02 (17–94)	38.80 ± 11.52	61.54 ± 11.98
Sex			
Male	299 (59.7%)	63 (12.6%)	236 (47.1%)
Female	202 (40.3%)	40 (8.0%)	162 (32.3%)
WHO Grade			
Grade 2	56 (11%)	46 (9%)	10 (2%)
Grade 3	43 (9%)	29 (5.8%)	14 (2.8%)
Grade 4	402 (80%)	28 (5.6%)	374 (74.65%)
MRI Sequences	T1c (IR-SPGR), T2w (3D FSE), FLAIR	Same	Same
Segmentation	Enhancing, necrotic, FLAIR abnormality regions	Same	Same

**Table 2:** The age, sex and IDH mutation distributions in the training and test datasets.

Feature	Centralized Train (n = 361)	Client 1 Train (n = 182)	Client 2 Train (n = 178)	Validation (n = 44)	Test (n = 59)
Age (years)	48.94 ± 18.13	58.61 ± 13.86	59.15 ± 14.10	54.23 ± 16.01	48.94 ± 18.13
Sex (% male)	57.50%	52.24%	57.87%	70.45%	61.02%
IDH Mutation Rate	15.55%	14.83%	16.29%	22.73%	50.84%

**Table 3:** Performance comparison of the proposed CL and FL approaches for identifying IDH mutation in gliomas in the test set.

Metric	Centralized (NSF *)	Centralized (GBSF **)	Federated Trimmed Mean (NSF *)	Federated Averaging Strategy (NSF *)
Accuracy	**0.949**	0.813	0.915	**0.949**
F1 Score	0.951	0.784	0.912	**0.952**
Precision	0.935	**0.952**	0.963	0.909
Recall	0.966	0.667	0.867	**1.000**
Specificity	0.931	**0.966**	**0.966**	0.896
ROC–AUC	**0.971**	0.907	0.957	0.967

* NSF: Naïve Soft Filtering; ** GBSF: Gradient-Based Soft Filtering; Bold values indicate the best performance for the corresponding metric.

## Data Availability

Publicly available datasets were analyzed in this study. All data used in this work are online datasets that can be accessed from the sources and repositories cited in the manuscript. No new datasets were generated.

## Abbreviations

HIPAA	Health Insurance Portability and Accountability Act
GDPR	General Data Protection Regulation
GBSF	Gradient-Based Soft Filtering
MR	Magnetic Resonance
NSF	Naive Soft Filtering
RGB	Red, Green, and Blue
ROI	Region-of-Interest
TI	Inversion Time
TE	Echo Time
TR	Repetition Time
IR-SPGR	Inversion-Recovery Spoiled Gradient Echo
UCSF-PDGM	University of California San Francisco Preoperative Diffuse Glioma MRI Dataset
FA	Federated Averaging
FTM	Federated Trimmed Mean
CL	Centralized Learning
FL	Federated Learning
DTI	Diffusion Tensor Imaging
ViT	Vision Transformer
FLAIR	Fluid-Attenuated Inversion Recovery
T1c/T1CE	T1-weighted Contrast-Enhanced
T1	T1-weighted
IDH	Isocitrate Dehydrogenase
WHO	World Health Organization
WHO CNS5	WHO Classification of Central Nervous System Tumors (5th Edition)
IDH-mut	IDH Mutant
IDH-wt	IDH-Wildtype
MRI	Magnetic Resonance Imaging
DL	Deep Learning
CNN	Convolutional Neural Network
